# Investigation of the Aquatic Photolytic and Photocatalytic Degradation of Citalopram

**DOI:** 10.3390/molecules26175331

**Published:** 2021-09-02

**Authors:** Cristina Jiménez-Holgado, Paola Calza, Debora Fabbri, Federica Dal Bello, Claudio Medana, Vasilios Sakkas

**Affiliations:** 1 Laboratory of Analytical Chemistry, Department of Chemistry, University of Ioannina, 45110 Ioannina, Greece; crijimhol@gmail.com (C.J.-H.); vsakkas@uoi.gr (V.S.); 2 Department of Chemistry, University of Torino, Via Giuria 5, 10125 Torino, Italy; paola.calza@unito.it; 3 Department of Molecular Biotechnology and Health Sciences, University of Torino, Via Giuria 5, 10125 Torino, Italy; federica.dalbello@unito.it (F.D.B.); claudio.medana@unito.it (C.M.)

**Keywords:** citalopram, photocatalytic degradation, transformation products, high-resolution mass spectrometry, toxicity, photodegradation

## Abstract

This study investigated the direct and indirect photochemical degradation of citalopram (CIT), a selective serotonin reuptake inhibitor (SSRI), under natural and artificial solar radiation. Experiments were conducted in a variety of different operating conditions including Milli-Q (MQ) water and natural waters (lake water and municipal WWT effluent), as well as in the presence of natural water constituents (organic matter, nitrate and bicarbonate). Results showed that indirect photolysis can be an important degradation process in the aquatic environment since citalopram photo-transformation in the natural waters was accelerated in comparison to MQ water both under natural and simulated solar irradiation. In addition, to investigate the decontamination of water from citalopram, TiO_2_-mediated photocatalytic degradation was carried out and the attention was given to mineralization and toxicity evaluation together with the identification of by-products. The photocatalytic process gave rise to the formation of transformation products, and 11 of them were identified by HPLC-HRMS, whereas the complete mineralization was almost achieved after 5 h of irradiation. The assessment of toxicity of the treated solutions was performed by Microtox bioassay (*Vibrio fischeri*) and in silico tests showing that citalopram photo-transformation involved the formation of harmful compounds.

## 1. Introduction

In the 2030 Agenda for Sustainable Development, adopted by UN Member States in 2015, one important goal is devoted to ensuring the availability and the sustainable management of water [1]. In this framework, the study concerning the fate of organic pollutants in the aquatic environment has the aim to assess if their release and the formation of by-products in the aqueous matrices can present a potential health hazard. These aspects should be taken into consideration in order to implement the most suitable strategies to abate pollutants or to restrict their discharge in the environment. Among the xenobiotic compounds that can have negative impacts on human health and aqueous ecosystems, the pharmaceuticals represent a class of greatest environmental concern due to their low degradability and their continuous inlet in the environmental waters [2,3].

Citalopram (CIT) is a selective serotonin reuptake inhibitor (SSRI) widely used in Northern Europe as antidepressant drug and it has been detected in influent, effluent, downstream and hospital wastewater treatment plants (WWTPs) at concentration levels up to about 300–800 ngL^−1^ [4,5]. There is a large range of half-lives on the literature for CIT depending on the pH of the sample, the matrices, the concentration of the pollutant and many other variables [6,7,8,9]. For example, J. Kwon et al. found a half-life of 65 days for citalopram in ultrapure water buffered at pH 9, of both 14 and 43 days in two different lakes and of 24 days in a WWTP sample under a fluorescent lamp [7]. R. Osawa et al. reported that citalopram was 100% degraded after 4 h under a UV lamp in a lake water sample [6]. The photodegradation of CIT was also reported to be induced by the natural organic matter (NOM) present in surface water [7]. Indeed, NOM that contains humified light-absorbing compounds can photoinduce the degradation of chemicals through the generation of reactive species such as excited triplet states (^3^DOM*) [10], hydroxyl radical (·OH) [11,12,13], singlet oxygen (^1^O_2_) [14,15], superoxide radical anion (O_2_^−^) and hydrogen peroxide (H_2_O_2_) [16].

CIT was categorized as a harmful substance with a value of EC50 equal to 30.14 mgL^−1^ on *Daphnia magna* (48 h exposure) without excluding possible chronic toxicity at lower exposure concentrations [17]. Recently, investigations about the effects of this antidepressant on the behavior of two different life stages of fish revealed that this drug poses potential risks for the aquatic environment and, therefore, its presence in water matrices has to be monitored carefully [18]. Previous studies showed that photolysis and hydrolysis do not significantly remove the molecule [19] and two of the transformation products (TPs) identified during the photolytic process were found also as human metabolites [7]. Taking into account that CIT was moderately removed by the aerobic and anoxic biodegradation [20], some advanced water treatment technologies (e.g., O_3_, ClO_2_ oxidation, UV irradiation and Fenton oxidation) have been applied to abate CIT [4] with a particular focus on the transformation products formed during the processes. In the case of the ozonization, the complete mineralization was not achieved in 90 min and the identified intermediates matched with those observed during the ClO_2_ oxidation, even if with different abundances.

Considering that, once released in the aqueous environment, pollutants can undergo biotic and abiotic degradation [5,21,22], this work aimed to investigate the photoinduced fate of CIT under simulated and solar irradiation in MilliQ water and in the presence of dissolved organic matter, bicarbonate and nitrate ions, as well in two real matrices (lake water and municipal WWT effluent). Moreover, in order to assess the possible application of the heterogenous photocatalysis to remove CIT in water, we performed irradiation experiments in the presence of titanium dioxide, one of the most widely exploited semiconductors to abate several classes of water pollutants [22,23,24]. Particular attention was focused on the identification of the transformation by-products formed during the process and on the evaluation of toxicity, performed by bioluminescent test (*Vibrio fischeri* bacterium) and in silico approach (US EPA ECOSAR computer model).

## 2. Results and Discussion

### 2.1. Irradiation Experiments

#### 2.1.1. Natural Sunlight Experiments

Experiments were carried out in MQ water as well as natural waters (lake and WWTP samples) at 10 mgL^−1^ of CIT. The quality parameters for the collected lake and WWTP samples are presented in Table S1 and Table S2. All the studied samples were characterized by a medium-to-high abundance of organic matter [25], with a NOM values of 23.2 and 11.7 mgL^−1^ for WWTP effluent and lake water, respectively. Nitrate in these waters was significantly higher in WWTP than in lake samples (86.1 and 10.5 mgL^−1^, respectively).

**Figure 1:**
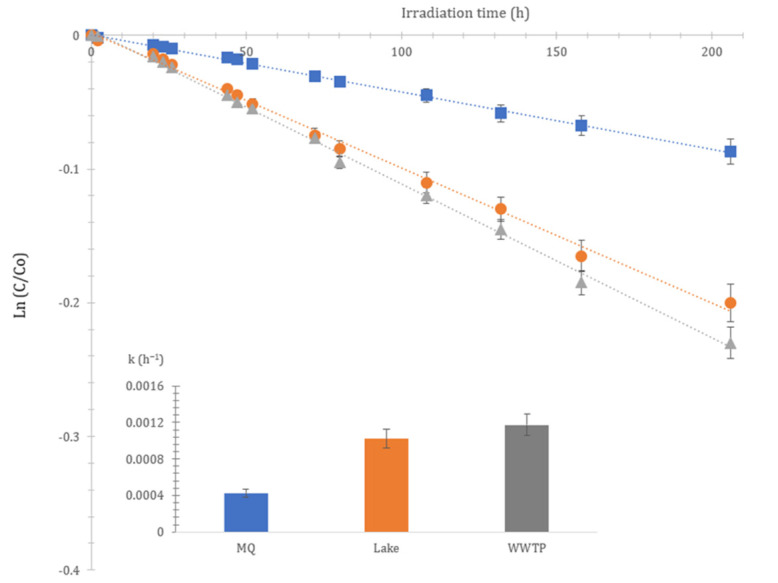
Kinetics and rate constant (h^−1^) of citalopram in different matrices under natural solar light.

Dark controls were found to be stable for all treatments, as less than 10% was degraded over the course of the exposure period. This hydrolytically stable behavior has been observed also by Kwon and Armbrust [7]. Considering the UV/Vis absorption spectrum of CIT, reported in a previous work [26], and the low absorbance above 290 nm, the direct photolysis rate in MQ water seems to be low with a half-live of 144 days (Figure 1 and Table 1) under the investigated conditions, revealing that CIT is a highly photostable molecule under these conditions.

On the other hand, the photodegradation rates of CIT in wastewater and lake water were significantly higher than in MQ water, thus showing that the aqueous matrix played a significant role in promoting the photolysis of CIT. When exposed to natural sunlight in wastewater, the CIT photodegradation rate was 2.8-fold faster compared to MQ water. This sensitization effect was observed for lake water as well. The CIT photodegradation rate was almost 2.4 times higher in lake water compared to MQ, indicating a photosensitization effect in the respective aqueous matrices. In order to confirm this relationship and to assess the contribution of different natural aqueous constituents, experiments at the same water matrices and also in the presence of NOM, nitrate and bicarbonate ions were carried out in laboratory-simulated irradiations.

#### 2.1.2. Simulated Solar Irradiation Experiments

**Figure 2:**
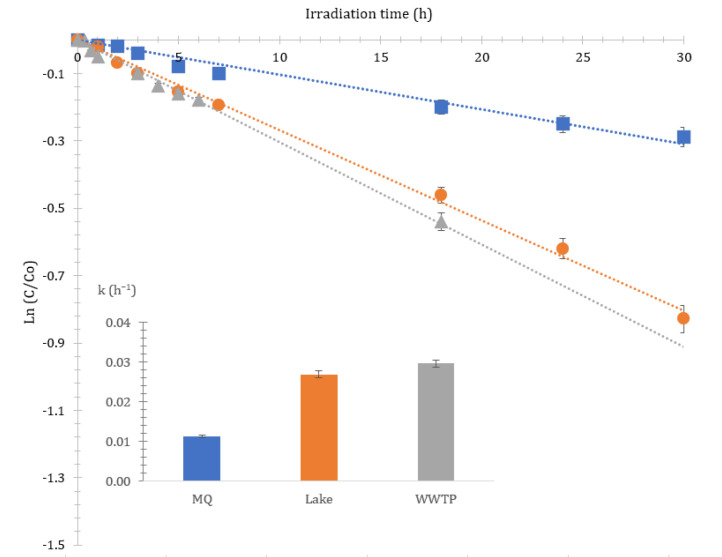
Kinetics and rate constant (h^−1^) of citalopram in different matrices under artificial solar irradiation.

Figure 2 depicts the degradation profiles of CIT and the photolysis rate constants in the three matrices. All degradation curves were well matched with a pseudo-first-order kinetic model with r^2^ generally above 0.94. The half-lives ranged between 62 h in MQ water to 23 h in WWTP effluent (see Table 1).

At the same conditions, no degradation was observed in dark controls in either MQ water or lake/WWTP samples, indicating that the compound under investigation was hardly susceptible to hydrolysis. Photolysis rates followed the same order as the natural sunlight experiments: WWTP > lake > MQ water, thus indicating that constituents of natural waters affect CIT’s decomposition [26]. According to the Table S1 and Table S2, the natural organic matter (NOM) concentration is higher in WWTP, followed by the lake, thus showing that, as the NOM concentration increases in natural waters, the rate of photolysis increases.

Hence, in order to further elucidate the role of organic matter on the performance of the photolytic process, Suwannee River natural organic matter was used as a representative of NOM at a concentration level of 10 mgL^−1^ in MQ water. A significant acceleration effect of the photolytic rate was observed. In a previous study we showed that water-extractable organic matter (WEOM) was able to produce an oxidant triplet excited state (^3^WEOM*), singlet oxygen (^1^O_2_) and hydroxyl radicals under irradiation with simulated solar light [11]. Apart from NOM, the effect of nitrate and bicarbonate ions on the photolytic rate was also investigated (Table 1). The results demonstrated that nitrate at 10 mgL^−1^ enhanced the photolytic rate of CIT, as well as bicarbonate (in the presence of 1 mgL^−1^ of nitrate), but to a lesser extent, in agreement with Kwon and Armbrust [7].

The results showed that the degradation order in artificial laboratory irradiation experiments are in good agreement with the experiments under natural sunlight, suggesting that laboratory-scale experiments using artificial irradiation can help predicting the behavior of CIT in natural waters [27]. However, the photodegradation rate of each case cannot be compared directly due to differences in the irradiation spectrum and daily light variation (night-day circle) [27,28]. Both of these experimental approaches should be viewed as complementary and as offering additional knowledge of the processes that occur in real waters by providing a better understanding of the photodegradation actually occurring in the natural environment [29,30].

Nevertheless, the time needed for the total elimination of the antidepressant in the natural environment seemed to be far from effective; therefore, the heterogeneous photocatalysis was assessed as an effective method of decontamination.

#### 2.1.3. Photocatalytic Experiments

**Figure 3:**
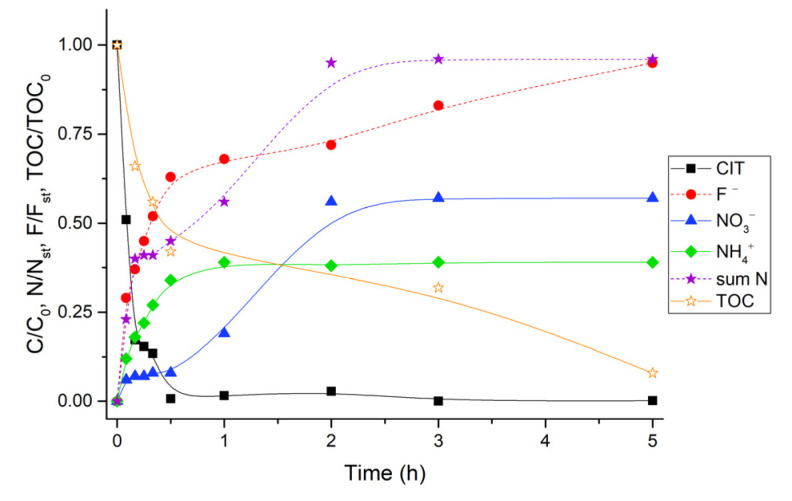
Time evolution of primary process, end-inorganic products (nitrate, ammonium and fluoride) and organic carbon during the photocatalytic degradation of CIT (CIT 20 mgL^−1^, TiO_2_ 400 mgL^−1^, the concentration of different species are normalized for the initial concentration (C/C_0,_ TOC/TOC_0_) or for the stoichiometric amount (N/N_st_ and F/F_st_); sum N represents the sum of nitrate and ammonium contribution).

As expected, the presence of the semiconductor accelerated the degradation of the pollutant. Under the adopted experimental conditions, the complete abatement of CIT by heterogenous photocatalysis occurred in around half hour (see Figure 3), whereas the organic carbon mineralization proceeded much slower than the primary process. About 50% of initial total organic carbon (TOC) content was mineralized within 1 h of irradiation, but only after 5 h did the TOC residual percentage reach a value <10%. The delay between the primary process and TOC evolution was attributed to the formation of TPs.

Nitrogen was released over time as nitrate and ammonium ions and the stoichiometric amount was achieved within 2 h. Ammonium was more easily released and reached a plateau value at 60 min, while nitrate ions exhibited a sharp increase later between 1 and 2 h. Taking into account that the cyano group is mainly transformed into nitrate [31], the evolution of this ion suggested that the oxidative process occurred on this part of molecule only at longer times of irradiation. The identification of several transformation products that still bear a carbonitrile substituent agreed with this hypothesis (see below). No traces of nitrite ions were detected under the employed experimental conditions. During the photocatalytic degradation, we also followed the evolution of fluoride arising from the breaking of bond C–F present in the molecular structure. Like the nitrogen trend, the fluoride concentration reached the 50% of the stoichiometric concentration within a half hour and then achieved the maximum after 5 h. 

### 2.2. Identification and Evolution of TPs

The HRMS analysis allowed us to identify and characterize 11 transformation products of CIT formed during the first steps of the photocatalytic process, and they are collected in Table 2 and Table S3. A level of confidence recommended by Schymanski et al. [32] was assigned to each TP and is reported in Table 2 as well. Although already present in the literature [6], the MS^2^ spectrum of CIT (*m*/*z* 325.1718) is reported in Figure S1 for the sake of clarity. Therein, it can be observed the fragment at *m*/*z* 307.1612 arising from the loss of water (H_2_O), together with the rearrangement of furan ring and the fragment at *m*/*z* 280.1139 due to the loss of dimethylamine. The detachment of both groups justifies the base peak at *m*/*z* 262.1032, whereas the fragment at *m*/*z* 109.0445 is consistent with the fluorotropylium ion formation.

Two signals at *m*/*z* 341.1669 with the chemical formula C_20_H_22_O_2_N_2_F were detected and attributed to mono-hydroxylated products (TP341A, B), where the OH attack was supposed to occur on the lateral chain.

The MS^2^ spectrum of TP341A (see Figure S2) suggests that the hydroxylation involved the lateral chain giving an intermediate already identified in the presence of organic matter [26] and during the UV and hydrolysis processes [6]. Indeed, the base peak at *m*/*z* 323.1554 shows that the loss of the first molecule of H_2_O is more favorable than that observed for CIT, as it does not entail the rearrangement of the furan ring that conversely can be involved to justify the second H_2_O loss (*m*/*z* 305.1455).

Regarding the TP341B, detected in this work for the first time, the MS^2^ spectrum reported in Figure S3 shows some peculiar differences. The abundance of the fragment at *m*/*z* 323.1560 and the absence of *m*/*z* 305.1449 are still consistent with the presence of a hydroxyl group on the chain, but in a position close to the furan ring, thus hindering the loss of a second molecule of H_2_O. The detachment of NH(CH_3_)_2_ and the further loss of H_2_O lead to the formation of the fragments at *m*/*z* 296.1088 and *m*/*z* 278.0981, respectively. The presence in the MS^2^ spectrum of a fragment corresponding to fluorotropylium (*m*/*z* 109.0444), also detected for CIT, excludes the hydroxylation involved the fluorophenyl moiety. However, the formation of TP339 *(vedi infra)* may suggest an OH attack on the furan ring, although in this case, the loss of a molecule of H_2_O should be less favored.

The intermediate TP357 at [M + H]^+^ 357.1618 and empirical formula C_20_H_22_O_3_N_2_F indicated the formation of a dihydroxylated product. This TP was identified here for the first time; it presents in its MS^2^ spectrum two main peaks only, attributable to the loss of two H_2_O molecules (see Figure S4), likely from the furan ring and the alkyl chain. Considering the overall degradation pathway, it was reasonable to assume that the hydroxylation involved both the furan ring and alkyl chain. 

Concerning the TP323 (*m*/*z* 323.1762), the elemental composition C_20_H_23_O_2_N_2_ has been proposed, consistent with the substitution of the fluorine atom by an OH group on the benzene ring bearing to the formation of a phenol structure. To support this statement and in agreement with the literature [4,26], the MS^2^ fragmentation pathway appears similar to that observed for CIT but with the formation of the hydroxytropylium ion (*m*/*z* 107.04888), as reported in Figure S5. 

The TP339 (*m*/*z* 339.1512 and chemical formula C_20_H_20_O_2_N_2_F) can be assigned to an oxidized product. The MS^2^ spectrum (Figure S6) matches well with the data reported in the literature [6,26]; the key fragment with *m*/*z* 248.0876 can be explained by the loss of a CO group, thus supporting the formation of a carbonyl group on the furan ring.

A transformation product with *m*/*z* 355.1457 and chemical formula C_20_H_20_O_3_N_2_F was detected (TP 355) and is consistent with the hydroxylation of TP339 [26]. The RDB for this intermediate was higher than that of CIT, also suggesting the formation of a carbonyl group for this by-product. The MS^2^ spectrum (see Figure S7) reports a fragment at *m*/*z* 337.1353 resulting from the loss of a molecule of H_2_O and the base peak at *m*/*z* 292.0775 arising by the joint loss of NH(CH_3_)_2_ and H_2_O. Considering the abundance of *m*/*z* 337.1353 (60%), we can hypothesize the presence of an OH on the alkyl chain that promotes this fragmentation. A further loss of the CO group involving the furan ring generates the signal at *m*/*z* 264.0825, thus confirming the presence of a carbonyl group. Finally, the fragment at *m*/*z* 274.0668 arises from the joint loss of two molecules of H_2_O, one of which entailed the furan ring, and of the dimethylamine group.

The analysis of MS^2^ spectrum of TP337 (Figure S8) suggested a dehalogenated structure bearing an OH group and a carbonyl group, as reported in the literature [6]. However, the fragment at *m*/*z* 319.1447 (−18 Da) is not very intense, indicating that, in this case, the loss of H_2_O involves the rearrangement of furan ring in the CIT spectrum and not from an OH group present on the chain. Furthermore, the peak base at *m*/*z* 274.0868 corresponds to the contemporaneous loss of one molecule of H_2_O and NH(CH_3_)_2_ group, whereas a further loss of H_2_O molecule is observed at very low intensity as it occurs on the fluorobenzene ring (*m*/*z* 256.1762). For the above-mentioned reasons, differently from the hypothesis of Osawa and co-workers [6], it may be assumed that the hydroxylation occurred on the benzene ring alongside the detachment of the fluorine atom, as already reported for the photocatalytic degradation of similar structures [33].

TP247 (*m*/*z* 247.1446 and chemical formula C_14_H_19_O_2_N_2_) is involved in the detachment of the fluorophenyl ring together with mono-hydroxylation. Unfortunately, the MS^2^ shows only one abundant fragment at *m*/*z* 229.1339 due to the loss of H_2_O (Figure S9), which is not useful to determinate the exact position of an OH attack. Taking into account the main mechanisms occurring in the photocatalytic degradation, it can be hypothesized that a substitution by an OH radical on the quaternary carbon that loses the aromatic ring, supported by the formation of unsaturated system in the fragment *m*/*z* 184.0757, which can prevent the rearrangement of furan ring and the loss of a second molecule of water.

Two isomers were detected with *m*/*z* 245.1292 and chemical formula C_14_H_17_N_2_O_2_, which result from the detachment of the fluorophenyl group, analogously to the TP247, and with the presence of a carbonyl group (Figure S10 and Figure S11). As observed for CIT and reported in literature [6], the MS^2^ spectrum of TP245B exhibits a fragment at *m*/*z* 227.1183 that can be explained by the loss of H_2_O and the most intense peak at *m*/*z* 200.0708 arising from the loss of the dimethylamine group. The signal at *m*/*z* 182.0602 is ascribable to the joint loss of H_2_O and NH(CH_3_)_2_ molecules, whereas the fragment at *m*/*z* 158.0236 with chemical formula C_9_H_4_O_2_N can be explained by the presence of the carbonyl group on the furan ring. As regards the TP245A, it can be hypothesized by a different position of the carbonyl group, as the MS^2^ spectrum shows only a fragment at *m*/*z* 200.0708. The absence of signals arising from the H_2_O loss can be justified by the involvement of the carbon in the chain in the α position in respect to the furan ring that prevented the rearrangement of molecule favoring only the loss of NH(CH_3_)_2_.

The TP261 with *m*/*z* 261.1241 and chemical formula C_14_H_17_O_3_N_2_ involves a further hydroxylation of TP245 (see Figure S12). The fragmentation pattern in MS^2^ suggests the formation of an intermediate similar to TP245B but with a further hydroxylation on the alkyl chain in agreement with the literature [6].The high abundance of the fragment at *m*/*z* 243.1132 corresponding to the loss of H_2_O suggests that the hydroxylation did not occur on the aromatic moiety, whereas the signal at *m*/*z* 216.659 is consistent with the detachment of NH(CH_3_)_2_. The concomitant loss of H_2_O and dimethylamine agreed with the fragment observed at *m*/*z* 198.0552 for which the chemical formula C_12_H_8_O_2_N was proposed. In contrast with photolytic process, no *N*-oxygenation and *N*-demethylation derivatives were detected during the photocatalytic treatment in the adopted experimental conditions.

**Scheme 1:**
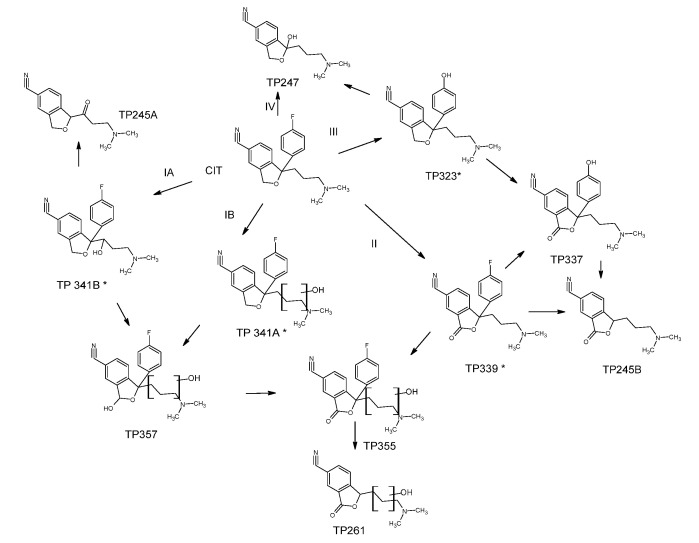
Proposed photocatalytic degradation pathway of CIT.

Based on MS data and the time evolution of TPs reported in Figure S13, a possible pathway for the early steps of CIT degradation is proposed in Scheme 1.

The photocatalytic degradation of CIT proceeds through the formation of mono- and di-hydroxylated intermediates (route IA and IB), where the OH attack involves primarily the alkyl chain and the furan ring producing the TP341A, B and T357.

Furthermore, the heterocycle structure can undergo oxidation to give the TP339 following the route II with the formation of a carbonyl group on the structure. The oxidized and hydroxylated intermediate TP355 can be yielded by pathways I and II, as it can be generated by hydroxylation of TP339 or oxidation of TP357.

The subsequent steps of degradation result in the detachment of the fluorophenyl group with the formation of TP261 and TP245B, the former arising from TP355, and the second from TP339 and TP337. The TP245A also shares a similar structure but it can be supposed to be produced from TP341B by a further oxidation of alcoholic group on the alkyl chain to a carbonyl group. The route III provides for the substitution reaction leading to the formation TP323 where the fluorine atom in the phenyl moiety is replaced by an OH group. Successively, the oxidation of this intermediate on the furan ring produces TP337 that can also be obtained through the route II via the dehalogenation/hydroxylation of TP339. Finally, the TP247 can be formed through the path IV or from TP323 by the substitution of the fluorophenyl moiety by the OH group.

Four of the identified intermediates in photocatalytic treatment were also detected during the direct photolysis in MQ and they have been marked in the Scheme 1 with an asterisk.

In photocatalytic experiment, TP341 A, B and TP339 were the most abundant and they achieved the maximum concentrations during the first few minutes of treatment. Then all transformation products of photocatalysis were totally degraded after 60 min, and the evolution profiles of TPs are shown in Figure S13.

### 2.3. Toxicity Assessment

**Figure 4:**
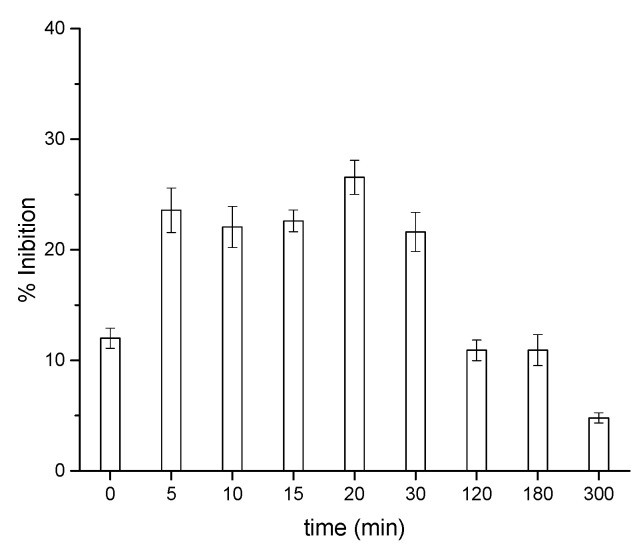
Acute toxicity of CIT as a function of the irradiation time in the presence of TiO_2_.

The acute toxicity trend during the treatment was assessed using the *Vibrio fischeri* assay (Figure 4). An increased percentage of inhibition was observed within the first stages of photocatalytic degradation reaching the maximum value of ca. 30% after 20 min, justified by the formation of transformation products that are moderately harmful toward bacteria. However, afterwards, the toxicity slowed down, settling at a negligible value (~5%) at the end of the treatment in agreement with the organic carbon content reduction described in Figure 3.

In a previous study, the in silico toxicity reported a higher toxicity for some of identified TPs during AOPs treatments towards *T. pyriformis* in respect to CIT and mutagenic and carcinogenic effects [6]. However, these previous results were affected by the type of text employed and did not consider the simultaneous presence of several TPs, so synergistic, antagonistic and additive effects cannot be excluded.

**Figure 5:**
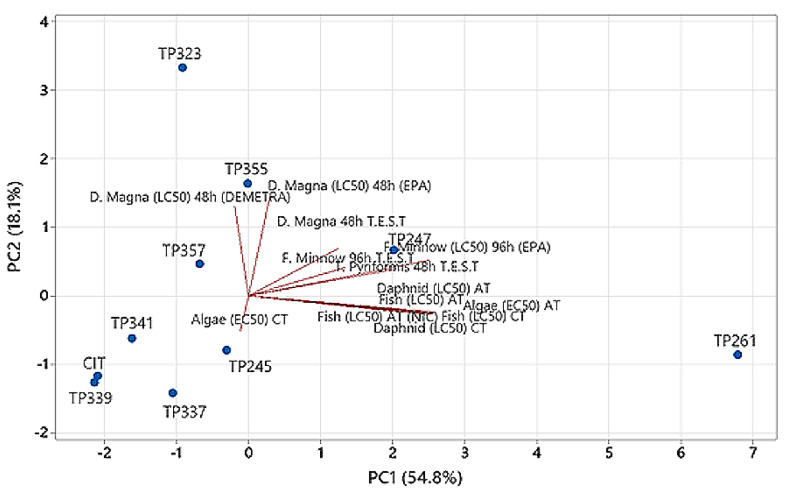
Comparison of aquatic toxicity of CIT TPs by PCA.

Furthermore, the latest versions of TEST and Vega software were employed along with ECOSAR to compare the aquatic toxicity results obtained by a luminous bacterium test. The evaluation of toxicity was estimated with the use of several models (Table 3), and therefore, principal component analysis (PCA) was applied to facilitate the interpretation of the information obtained (Figure 5).

In total, 13 models were applied, namely, acute for Daphnid LC50 48 h, Fish LC50 96 h and Green Algae EC50 96 h, and chronic for Daphnid LC50, Fish LC50 Green Algae EC50, (by ECOSAR), *D. magna* LC50 48 h, *F. minnow* LC50 96 h, *T. pyriformis* IGC50 48 h (by T.E.S.T.), *D. magna* LC50 48 h DEMETRA, Fish Acute NIC model and *F. minnow* LC50 96 h EPA model (Vega software). Raw data concerning aquatic toxicity are presented in Table 3. Based on the PCA calculations (Figure 5), the applied models were not strongly correlated (PC1 and PC2 explained 54.8% and 18.1% of the total data variance, respectively). The ECOSAR, T.E.S.T. and *F. minnow* LC50 96 h EPA models provided rather similar results in the same direction. The other models predicted a zero correlation since the angle between vectors was close to 90°. Thus, *D. magna* LC50 48 h EPA and LC50 48 h DEMETRA, or −90° Algae (EC50) CT. TP339, TP341 A-B, TP245 A-B, TP337 and TP357, were grouped along with the parent compound in a cluster. TP339 was more toxic than the parent molecule in 9 of the 13 models, while TP261 was found to be the less toxic compound (Table 3). Our findings support that irradiation treatment of pharmaceuticals can result in compounds that are more harmful than the parent substance [34].

## 3. Materials and Methods

### 3.1. Reagents and Materials

Citalopram (1-[3-(dimethylamino)propyl]-1-(4-fluorophenyl)-3*H*-2-benzofuran-5-carbonitrile, hydrobromide), with a purity grade >95%, was purchased from LGC Promochem (Molsheim, France). For analytical purposes, citalopram (CIT) was dissolved in methanol to provide a stock solution containing 1000 mg L^−1^ of analyte. The solution was stored in glass-stopped bottles at −20 °C in the dark. Standard working solutions were prepared daily. Ultrapure water used was produced by a Milli-Q system (Evoqua, Pittsburgh, PA, USA). Photosensitizers included freeze-dried Suwannee River natural organic matter (NOM), which was obtained from the International Humic Substances Society (Denver, CO, USA), and NaNO_3_ and NaHCO_3_ were purchased from Sigma-Aldrich (Athens, Greece). Irradiation procedures of CIT were carried out by experimental solutions that were prepared by dissolving CIT directly in Milli-Q water (MQ), MQ water + sensitizers, WWTP effluent and lake water. Acetonitrile (≥99.9%) was purchased from Merck Life Science S.r.l. (Milan, Italy). TiO_2_ P25 (Evonik Industries, Essen, Italy) was used as photocatalyst.

### 3.2. Sample Collection

Effluent and lake water samples of 2.5 L were collected from the municipal WWTP of Ioannina city and Pamvotis Lake, respectively, located in northwest Greece, in the region of Epirus. After collection, the samples were transported to the lab and were immediately filtered with 0.45 μm polyamide filters. The filtered samples were then kept in the dark at −5 °C until the irradiation experiments. The most relevant physicochemical characteristics of the aqueous samples are provided in Table S1.

### 3.3. Irradiation Procedures

#### 3.3.1. Natural Sunlight Irradiation

Experiments were carried out in the roof terrace of the Department of Chemistry of Ioannina University (2018) in MQ water as well as natural waters (WWTP effluent and lake water) at 10 mg L^−1^ of CIT. The solutions (1 L) were put into capped Pyrex glass bottles and were homogenized by magnetic stirring. Irradiation of the samples was conducted on platform with an inclination of 30° from the horizon, facing due south, following the recommendations of the Environmental Protection Agency. Dark control experiments were also performed by exposing amber glass bottles at the same concentration and covered with aluminum foil in the same environmental conditions in order to calculate for other degradation processes like hydrolysis and biodegradation. Aliquots (1.0 mL) were withdrawn from the bottles at specific time intervals. The average total daily short-wave radiation was 286 and 273 W/m^2^ for July and August, respectively, while the sunshine duration from sunrise to sunset was 10 and 11 h for the two months, respectively. The mean daily air temperature was 23 and 26 °C, with minimum air temperature at 17 and 18 °C and maximum air temperature at 26 and 32 °C for July and August, respectively. Experiments were carried out in duplicate.

#### 3.3.2. Simulated Solar Irradiation

Photodegradation experiments were also carried out at the same aqueous matrices and CIT concentration in a sunlight simulator (Solarbox, CO.FO.Me.Gra, Milan, Italy) equipped with a xenon lamp (1500 W) with a cut-off filter below 340 nm. On top of the solutions, the irradiance was 18 W m^−2^ in the 295–400 nm range (like the natural sunlight UV irradiance at middle European latitude on sunny days) [35]. Irradiation was carried out in magnetically stirred cylindrical closed cells (Pyrex glass, 40 mm i.d. 25 mm) on 5 mL aqueous solutions containing 10 mgL^−1^ of CIT, which were subjected to different irradiation times.

To test whether NOM, nitrate and bicarbonate ions (10 mgL^−1^ each) affect the photodegradation rate of CIT solutions (1 mgL^−1^), MQ water samples containing the above sensitizers were exposed to the same irradiation conditions. All experiments were carried out in triplicate including dark controls that were performed under the same conditions.

The rate constants were calculated by linear regression analysis of the plot of the first-order equation C_t_ = C_o_e^−kt^, where C_o_ is the initial concentration, C_t_ is the concentration at a certain time and k is the rate constant. The half-life is calculated by t_1/2_ = ln2/k.

#### 3.3.3. Photocatalytic Experiments

Photocatalytic experiments were performed in the same cylindrical closed cells on 5 mL aqueous solutions containing 20 mgL^−1^ of CIT and 400 mgL^−1^ TiO_2_. Samples were subjected to different irradiation times (ranging from 5 min to 3 h) using a Philips Cleo 6X15 W TL-D Actinic BL. After illumination, the samples were filtered through 0.45 μm Millex LCR hydrophilic PTFE membranes (Millipore) before the analysis. The lamp irradiance over the irradiated solutions was around 90 Wm^−2^ in the wavelength range of 290−400 nm (measured with a power meter from CO.FO.ME.GRA., Milan, Italy).

### 3.4. Analytical Procedures

Concentrations of CIT under all irradiation conditions was monitored with a Merck-Hitachi chromatograph equipped with Rheodyne injector (20 μL sample loop), L-6200 and L-6200A pumps for high-pressure gradients, L-4200 UV−vis detector and RP-C18 column (Lichrospher, 4 mm i.d. × 125 mm length, from Merck). Isocratic elution (1 mL·min^−1^ flow rate) was carried out with 80% of 0.01 M phosphoric acid solution at pH 2.8 and 20% acetonitrile. The detector wavelength was 239 nm.

The formation of ionic products in samples irradiated was followed by a suppressed ion chromatography, employing a Dionex DX 500 instrument (Thermo Scientific, Waltham, MA, USA) equipped with a conductometer detector. The anions (nitrate and nitrite) were separated by an AS9HC anionic column (200 mm long × 4 mm i.d.) using an aqueous solution of K_2_CO_3_ (9 mM) as mobile phase; the elution was performed at 30 °C at a flow rate of 1 mL·min^−1^. The determination of ammonium ions was performed by employing a CS12A column, using 25 mM methanesulfonic acid as the eluent at a flow rate of 1 mL·min^−1^.

The evolution of the total organic carbon (TOC) during the photocatalytic runs was followed using a using a Shimadzu TOC-V CSH (catalytic oxidation on Pt at 680 °C) equipped with a Shimadzu ASI-V autosampler. Calibration was achieved by injection of known amounts of potassium phthalate.

### 3.5. Identification of By-Products

HPLC-HRMS was used to determinate and elucidate CIT and its by-products under photocatalytic conditions. The chromatographic separations, monitored using an MS analyzer, were carried out with a Phenomenex Gemini NX C18 150 × 2.1 mm × 3 μm particle size (Phenomenex, Bologna, Italy), using an Ultimate 3000 HPLC instrument (Dionex, Thermo Scientific, Milan, Italy). The injection volume was 20 μL and the flow rate was 200 μL·min^−1^. The following gradient mobile phase composition was adopted: A/B was varied from 5/95 to 50/50 in 30 min, followed by second step to reach 100/0 in 5 min where A = formic acid 0.05% *v*/*v* in acetonitrile and B = formic acid 0.05% *v*/*v* in water. A LTQ Orbitrap mass spectrometer (Thermo Scientific, Milan, Italy) equipped with an ESI ion source was used. The LC column effluent was delivered into the ion source using nitrogen as both the sheath and auxiliary gas. The capillary voltage and tube lens voltage in the ESI source were maintained at 28 V and 70 V, respectively. The source voltage was set to 4.5 kV (in positive ion mode). The capillary temperature was maintained at 270 °C. The acquisition method used was optimized beforehand in the tuning sections for the parent compound (capillary, magnetic lenses and collimating octapole voltages) to achieve maximum sensitivity. The mass accuracy of the recorded ions (vs. calculated) was ±5 ppm, without internal calibration. Analyses were run using full scan MS (50–1000 *m*/*z* range), MS^2^ acquisition in the positive ion mode, with a resolution of 30,000 (500 *m*/*z* FWHM) in FTMS (full transmission) mode. The ions submitted to MS^2^ acquisition were chosen on the base of full MS spectra abundance without using the automatic dependent scan. The collision energy was set to 30% for all the MS^2^ acquisition methods. The MS^2^ acquisition range was between the values of the ion trap cut-off and *m*/*z* of the [M + H]^+^ ion. Xcalibur (Thermo Scientific, Milan, Italy) software was used both for acquisition and data analysis.

### 3.6. Toxicity Measurements

The toxicity samples collected at different irradiation times were evaluated with a Microtox Model 500 Toxicity Analyzer (Milan, Italy). Acute toxicity was determined with a bioluminescence inhibition assay using the marine bacterium *Vibrio fischeri* by monitoring changes in the natural emission of the luminescent bacteria when challenged with toxic compounds. Freeze-dried bacteria, reconstitution solution, diluent (2% NaCl) and an adjustment solution (non-toxic 22% sodium chloride) were obtained from Azur (Milan, Italy). Samples were tested in a medium containing 2% sodium chloride, in five dilutions, and luminescence was recorded after 5, 15 and 30 min of incubation at 15 °C. Since no substantial change in luminescence was observed between 5 and 30 min, the results related to 15 min of contact are reported below. Inhibition of luminescence, compared with a toxic-free control to give the percentage inhibition, was calculated following the established protocol using the Microtox calculation program.

Moreover, since the identified TPs of CIT are not commercially available, three different softwares were employed, namely ECOSAR (v. 2.0), the Toxicity Estimation Software Tool or T.E.S.T. (v. 5.1.1) and Vega (v. 1.1.5).

## 4. Conclusions

The photolytic behavior of the antidepressant drug CIT was investigated under both natural and simulated solar irradiation conditions, and its removal from water exploiting heterogenous photocatalysis was evaluated. Direct photolysis of CIT proceeds very slowly; however, indirect photolytic processes play a significant role on its environmental attenuation the aqueous matrices. As expected, the use of TiO_2_ greatly increased the degradation rate of CIT and brought about the formation of several TPs. Eleven transformation products were identified and characterized by means of high-resolution mass spectrometry. The complete abatement of CIT occurred at around 30 min, whereas the organic carbon mineralization processed in a much slower rate. Toxicity studies have shown that a few transformation products (mainly TP339) are more toxic than the parent molecule; however, the TiO_2_-based photocatalysis has been demonstrated to have a good efficiency to remove them completely.

## Figures and Tables

**Table 1:** Kinetics of the photolysis of citalopram in different aqueous media under solar and simulated solar irradiation.

Photolysis	t_1/2_ (h)	k (h^−1^)	*R* ^2^
Solar Irradiation (Outdoor)			
MQ water	3465.74	0.0004	0.9461
Lake Pamvotis	693.15	0.0010	0.9805
WWTP water	462.10	0.0012	0.9821
Simulated solar irradiation (Solarbox)			
MQ water	61.89	0.0112	0.9486
Lake Pamvotis	25.77	0.0269	0.9940
WWTP water	23.42	0.0296	0.9811
NOM 10 mg L^−1^	14.03	0.0494	0.9783
NO_3_^−^ 10 mg L^−1^	45.60	0.0152	0.9409
HCO_3_^−^ 10 mg L^−1^	27.08	0.0256	0.9604

**Table 2:** CIT and its TPs observed during the photocatalytic process.

Name, [M + H]^+^	Empirical Formula	Δppm	RT (min)	Confidence Level
Citalopram-325.1718	C_20_H_22_ON_2_F	2.251	19.92	L1
TP245A-245.1292	C_14_H_17_O_2_N_2_	3.042	6.54	L3
TP245B-245.1292	C_14_H_17_O_2_N_2_	3.042	8.41	L2a
TP247-247.1446	C_14_H_19_O_2_N_2_	2.005	5.79	L3
TP261-261.1241	C_14_H_17_O_3_N_2_	2.800	8.13	L3
TP323-323.1762	C_20_H_23_O_2_N_2_	2.462	14.16	L2a
TP337-337.1554	C_20_H_21_O_3_N_2_	2.168	13.93	L2b
TP339-339.1512	C_20_H_20_O_2_N_2_F	2.558	18.92	L2a
TP341A-341.1669	C_20_H_22_O_2_N_2_F	2.689	17.40	L3
TP341B-341.1669	C_20_H_22_O_2_N_2_F	2.689	18.70	L3
TP355-355.1457	C_20_H_20_O_3_N_2_F	1.275	17.84	L3
TP357-357.1618	C_20_H_22_O_3_N_2_F	2.528	16.42	L3

**Table 3:** Aquatic toxicity of CIT and its TPs (mg L^−1^).

	Fish (LC50) AT	Daphnid (LC50) AT	Algae (EC50) AT	Fish (LC50) CT	Daphnid (LC50) CT	Algae (EC50) CT	*F. minnow* 96 h T.E.S.T	*D. magna* 48 h T.E.S.T	*T. pyriformis* 48 h T.E.S.T	Fish (LC50) AT (NIC)	*F. minnow* (LC50) 96 h (EPA)	***D. magna*** **(LC50) 48 h (EPA)**	***D. magna*** **(LC50) 48 h (DEMETRA)**
**CIT**	4.47	0.652	0.36	0.14	0.065	0.14	1.2	0.34	7.99	1.06	2.92	0.0084	2.26
**TP245A, B**	260	27.2	29.1	22.7	1.97	8.8	4.2	1.26	27.1	62.9	12.38	0.0593	1.41
**TP247**	589	57.8	70	61.9	3.94	20.2	16.75	1.58	90.1	63.4	29.23	0.1288	11.44
**TP261**	2840	248	379	427	15.1	101	5.62	3.36	66.57	67.03	48.58	0.1326	5.54
**TP337**	294	31.2	32	24.2	2.29	988	2.29	2.57	12.31	0.4955	7.35	0.0187	2.33
**TP323**	61.6	7.35	6.06	3.58	0.604	2.01	3.24	2.65	20.76	1.06	23.93	0.4744	80.03
**TP339**	21.3	2.77	1.93	0.954	0.247	0.68	0.59	0.36	5.54	0.0835	1.63	0.0068	1.04
**TP341A, B**	48	5.86	4.62	2.6	0.493	1.56	2.29	1.75	16.64	0.5014	5.73	0.0195	6.49
**TP355**	249	26.9	27	19.7	2.01	8.35	5.25	4.7	42.33	0.0879	20.07	0.336	10.1
**TP357**	279	29.8	30.5	22.5	2.21	9.36	3.01	0.94	43.23	0.0879	13.44	0.2764	12.09

## Data Availability

Not available.

## Supplementary Materials

The following are available online. Table S1: Physicochemical properties of Lake Pamvotis, Table S2: Physicochemical characteristics of WWTP effluent aqueous sample, Table S3: MS^2^ product ions formed from CIT and its transformation products, Figure S1: MS^2^ spectrum of citalopram, Figure S2: MS^2^ spectrum of TP341A, Figure S3: MS^2^ spectrum of TP341B, Figure S4: MS^2^ spectrum of TP357, Figure S5 MS^2^ spectrum of TP323, Figure S6: MS^2^ spectrum of TP339, Figure S7: MS^2^ spectrum of TP355, Figure S8: MS^2^ spectrum of TP337, Figure S9: MS^2^ spectrum of TP247, Figure S10: MS^2^ spectrum of TP245A, Figure S11: MS^2^ spectrum of TP245B, Figure S12: MS^2^ spectrum of TP261, Figure S13: Time evolution of TPs detected during the photocatalytic treatment.

## Sample Availability

Not available.
