# Supplementary material for: Investigation of the Aquatic Photolytic and Photocatalytic Degradation of Citalopram
Source: Molecules. 2021 Sep 2;26(17):5331. doi: 10.3390/molecules26175331 (PMC8434266; doi:10.3390/molecules26175331)
Supplement: Supplementary file 1 [file molecules-26-05331-s001.zip › molecules-1330555-supplementary.pdf]

Supplementary material for

Investigation of the Aquatic Photolytic and Photocatalytic Degradation of Citalopram

Cristina Jiménez-Holgado [1], Paola Calza [2], Debora Fabbri [2], Federica Dal Bello[3], Claudio Medana [3], Vasilios Sakkas [1]

[1] Laboratory of Analytical Chemistry, Department of Chemistry, University of Ioannina, Ioannina 45110, Greece
[2] Department of Chemistry, University of Torino, Via Giuria 5, 10125 Torino, Italy;
[3] Department of Molecular Biotechnology and Health Sciences, University of Torino, Via Giuria 5, 10125 Torino, Italy.

Table S1. Physicochemical properties of Lake Pamvotis.

Parameter	Value
pH	8.3
Conductivity (μS cm^{-1})	262
TDS (mg L^{-1})	2213
NOM (mg L^{-1})	11.7
NO_3^- (mg L^{-1})	10.5
Br^- (mg L^{-1})	0.38
NO_2^- (mg L^{-1})	0.010
Cl^- (mg L^{-1})	88.0
HPO_4^{2-} (mg L^{-1})	0.03
NH_4^+ (mg L^{-1})	0.21

TDS: Total Dissolved Solids; NOM: Natural Organic Matter

Table S2 Physicochemical characteristics of WWTP effluent aqueous sample.

	Effluent of WWTP
pH	7.4
NOM[a] (mg L^{-1})	23.2
NO_3^-(mg L^{-1})	86.1
P total (mg L^{-1})	1.0
N total (mg L^{-1})	13.5

[a] NOM: natural Organic Matter

Table S3 MS^2 product ions formed from CIT and its transformation products.

$[M+H]^+$ and empirical formula	Δppm	RDB[a]	RT (min)	MS^2	Relative intensity (%)	Δppm	Rif.	
**Citalopram** 325.1718 $C_{20}H_{22}ON_2F$	2.251	11	19.92	307.1611 $C_{20}H_{20}N_2F$	13	1.942	[1]	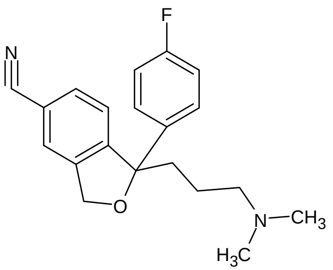
				280.1137 $C_{18}H_{15}ONF$	17	1.718		
				262.1031 $C_{18}H_{13}NF$	100	1.701		
				234.0718 $C_{16}H_9NF$	5	1.905		
				166.0651 $C_{12}H_8N$	3	-0.156		
				109.0444 C_7H_6F	23	-3.714		
**T245-A** 245.1292 $C_{14}H_{17}O_2N_2$	3.042	8	6.54	200.0708 $C_{12}H_{10}O_2N$	100	0.974	In this work	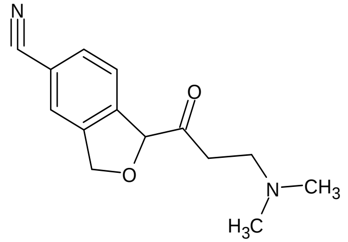
**T245-B** 245.1292 $C_{14}H_{17}O_2N_2$	3.042	8	8.41	227.1183 $C_{14}H_{15}ON_2$	20	1.807	[1]	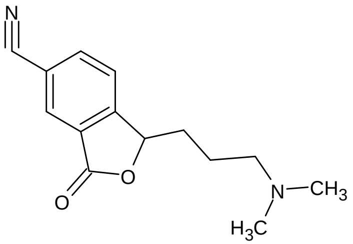
				200.0708 $C_{12}H_{10}O_2N$	50	0.974		
				182.0602 $C_{12}H_8ON$	16	0.876		
				158.0236 $C_9H_4O_2N$	20	-0.348		
**T247** 247.1446 $C_{14}H_{19}O_2N_2$	2.005	7	5.79	229.1339 $C_{14}H_{17}ON_2$	100	1.572	In this work	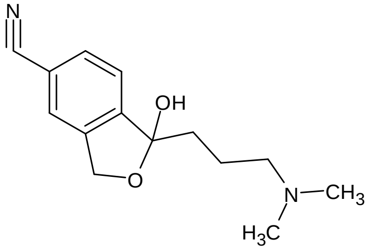
				202.0865 $C_{12}H_{12}O_2N$	2	1.212		
				184.0758 $C_{12}H_{10}ON$	5	0.595		

**T261** 261.1241 $C_{14}H_{17}O_3N_2$	2.800	8	8.13	243.1132 $C_{14}H_{15}O_2N_2$	90	1.628	[1]	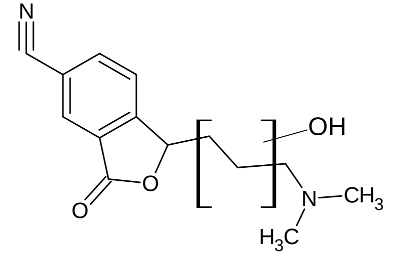
				216.0659 $C_{12}H_{10}O_3N$	100	1.760		
				198.0552 $C_{12}H_8O_2N$	30	1.237		
**T323** 323.1762 $C_{20}H_{23}O_2N_2$	2.462	11	14.16	305.1655 $C_{20}H_{21}ON_2$	10	2.163	[2][3]	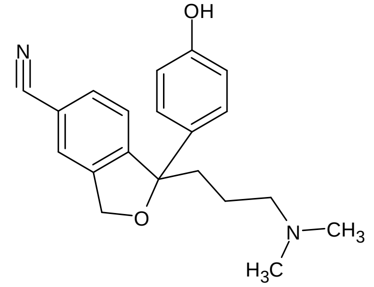
				278.1182 $C_{18}H_{16}O_2N$	20	2.318		
				260.1075 $C_{18}H_{14}ON$	100	1.958		
				242.0970 $C_{18}H_{12}N$	2	2.371		
				232.0762 $C_{16}H_{10}ON$	4	2.195		
				166.0652 $C_7H_7N_4F$	3	0.447		
				107.0488 C_7H_7O	15	-3.189		
**T337** 337.1554 $C_{20}H_{21}O_3N_2$	2.168	12	13.93	319.1447 $C_{20}H_{19}O_2N_2$	15	1.866	In this work	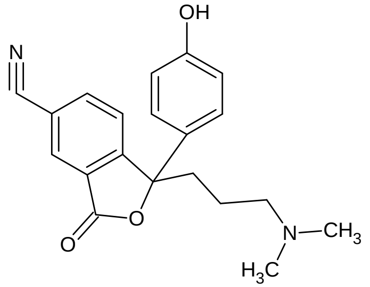
				292.0974 $C_{18}H_{14}O_3N$	15	1.986		
				274.0868 $C_{18}H_{12}O_2N$	100	1.988		
				256.0762 $C_{18}H_{10}ON$	15	1.990		
				172.0394 $C_{10}H_6O_2N$	10	0.552		
**T339** 339.1512 $C_{20}H_{20}O_2N_2F$	2.558	12	18.92	321.1406 $C_{20}H_{18}ON_2F$	5	2.591	[1][2][3]	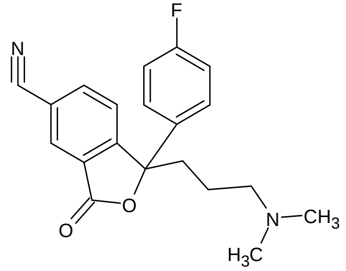
				294.0932 $C_{18}H_{13}O_2NF$	5	2.437		
				276.0825 $C_{18}H_{11}ONF$	40	2.105		
				258.0719 $C_{18}H_9NF$	5	2.115		

**T341 A** 341.1669 $C_{20}H_{22}O_2N_2F$	2.689	11	17.40	323.1557 $C_{20}H_{20}ON_2F$	100	0.870	[1][3]	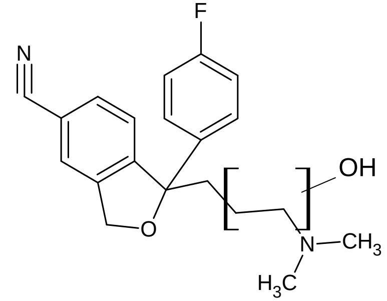
				305.1455 $C_{20}H_{18}N_2F$	42	2.119		
**T341 B** 341.1669 $C_{20}H_{22}O_2N_2F$	2.689	11	18.70	323.1560 $C_{20}H_{20}ON_2F$	100	1.801	In this work	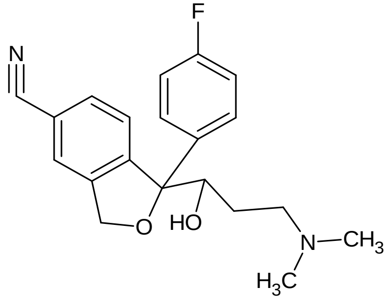
				296.1088 $C_{18}H_{15}O_2NF$	20	2.251		
				278.0981 $C_{18}H_{13}ONF$	70	1.910		
				240.0824 $C_{15}H_{11}ONF$	10	2.005		
				109.0444 C_7H_6F	6	-3.714		
**T355** 355.1457 $C_{20}H_{20}O_3N_2F$	1.275	12	17.84	337.1353 $C_{20}H_{18}O_2N_2F$	50	1.861	[3]	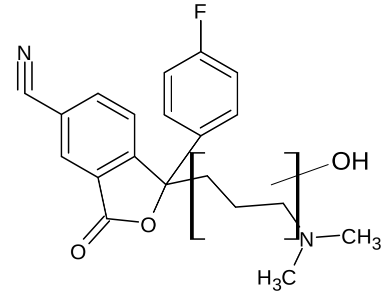
				292.0775 $C_{18}H_{11}O_2NF$	100	2.283		
				274.0668 $C_{18}H_9ONF$	13	1.939		
**T357** 357.1618 $C_{20}H_{22}O_3N_2F$	2.528	10.5	16.42	339.1509 $C_{20}H_{20}O_2N_2F$	100	1.673	In this work	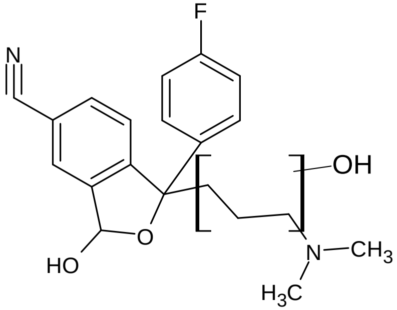
				321.1404 C_{20} $H_{18}ON_2F$	17	1.968		

[a] RDB was calculated for uncharged species.

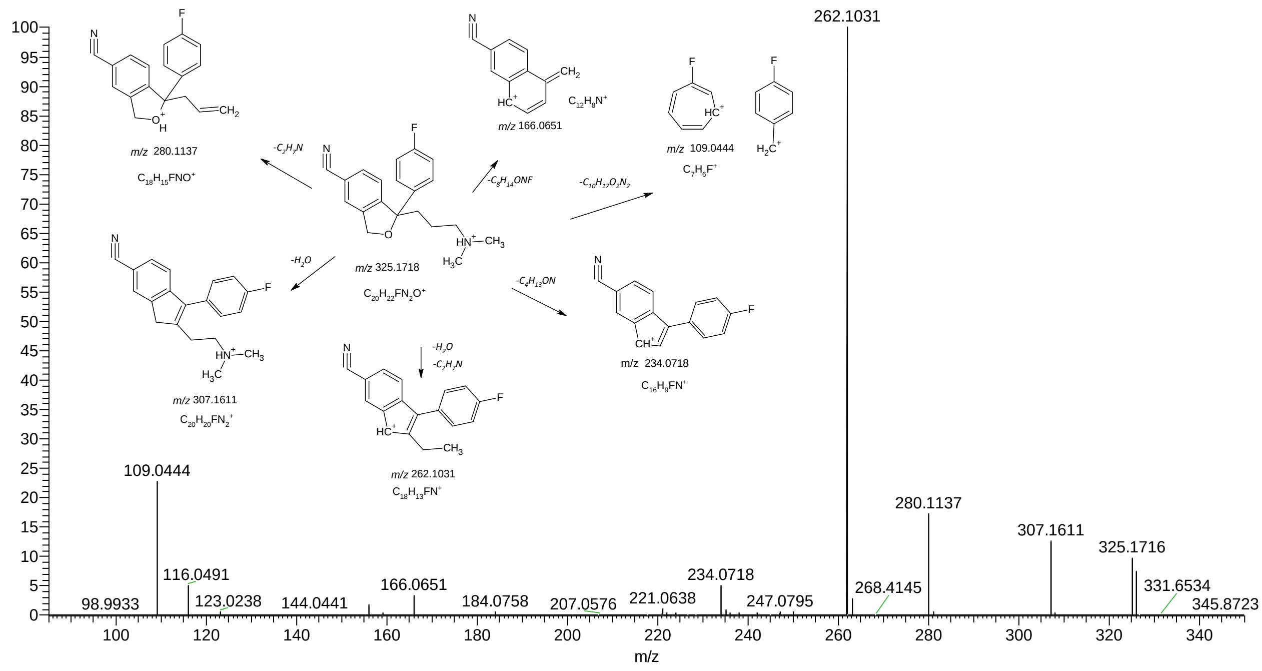

Figure S1 MS^2 spectrum of Citalopram.

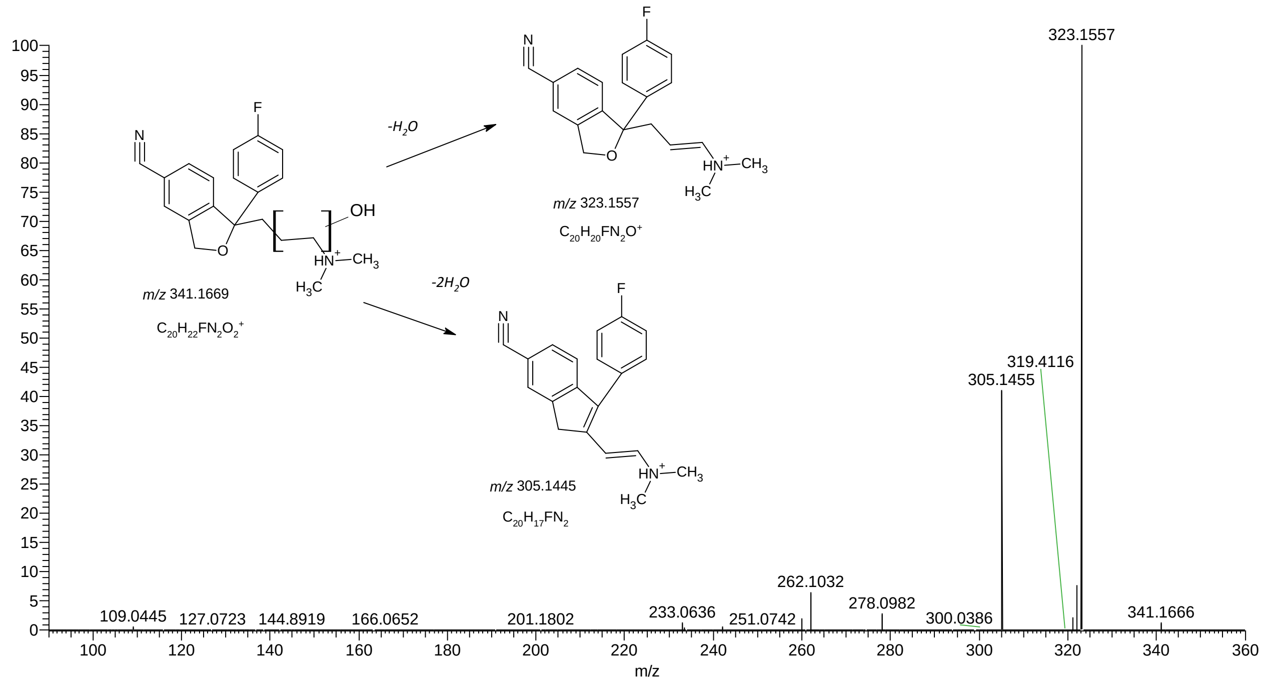

Figure S2 MS^2 spectrum of **TP341A.**

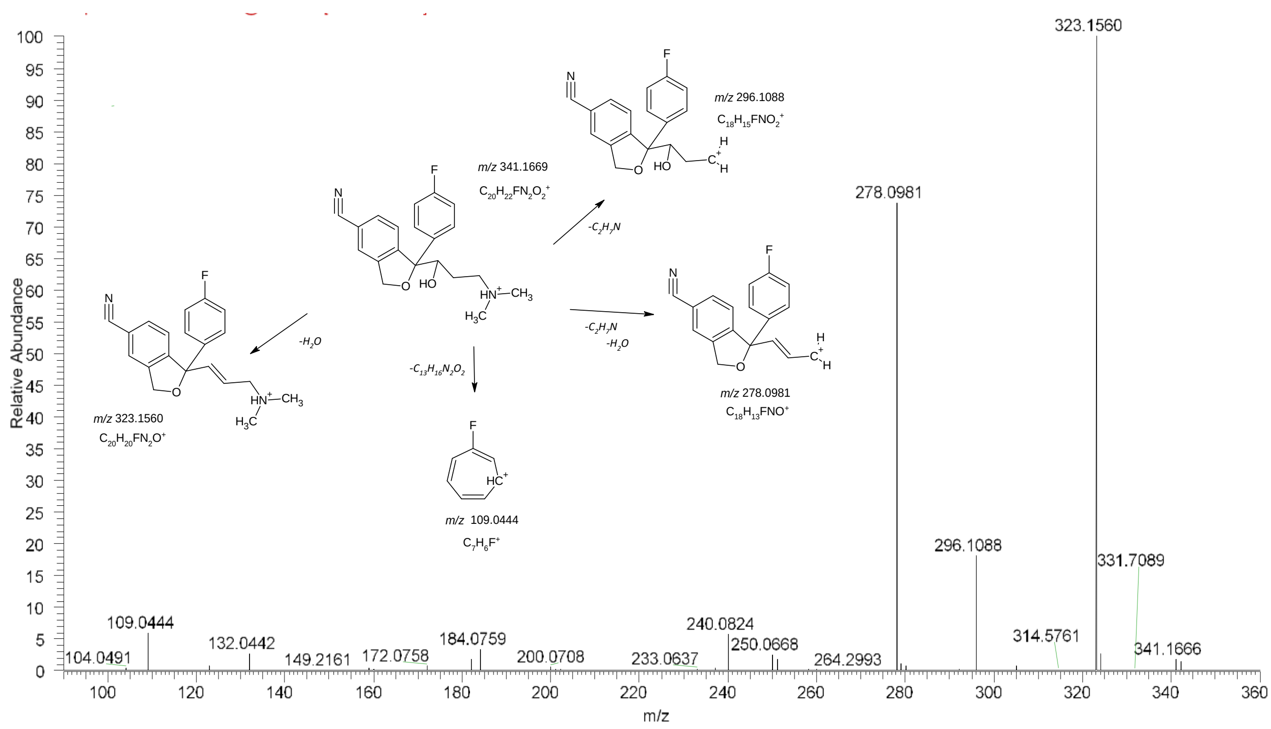

Figure S3 MS^2 spectrum of **TP341B.**

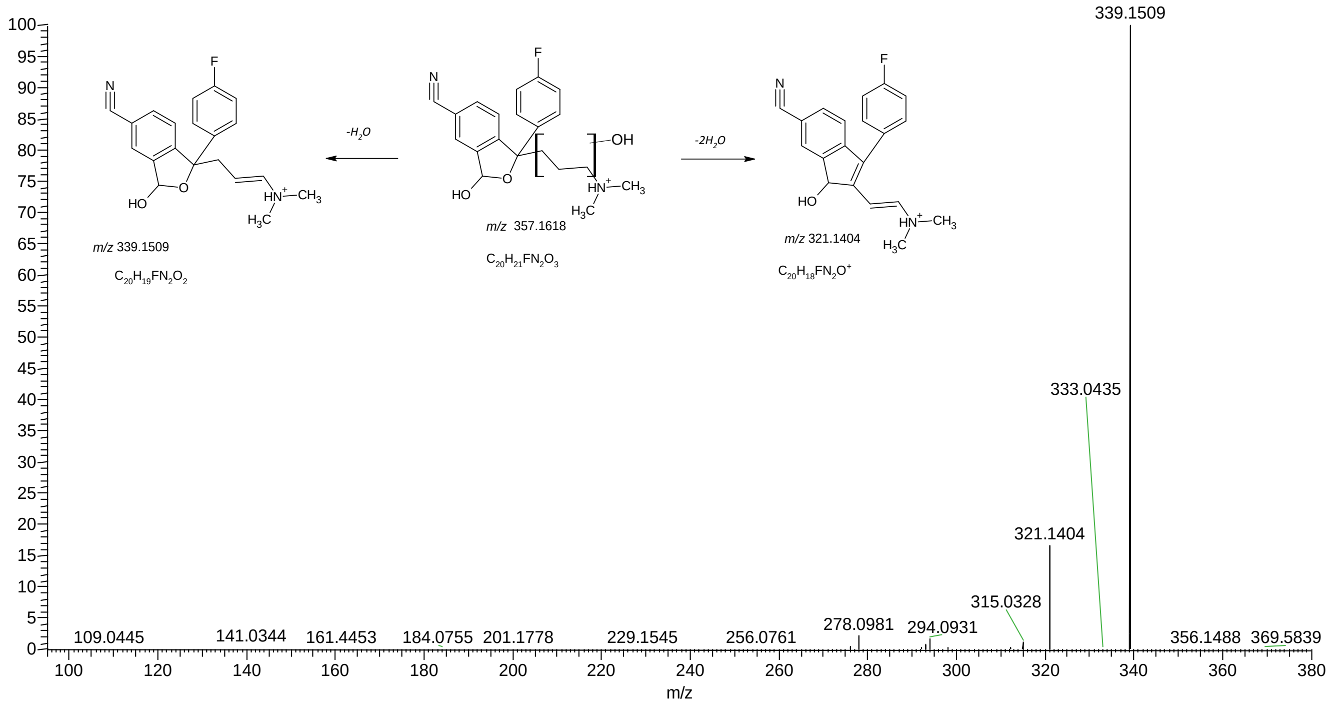

Figure S4 MS^2 spectrum of **TP357.**

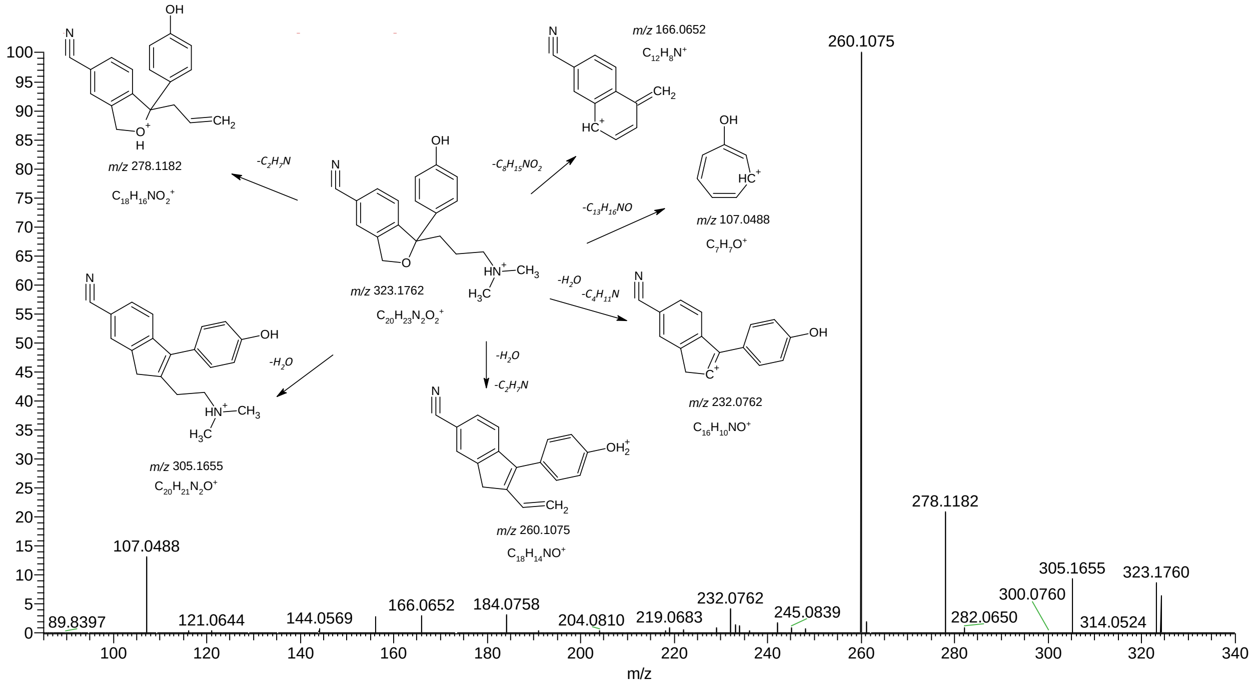

Figure S5 MS^2 spectrum of **TP323**.

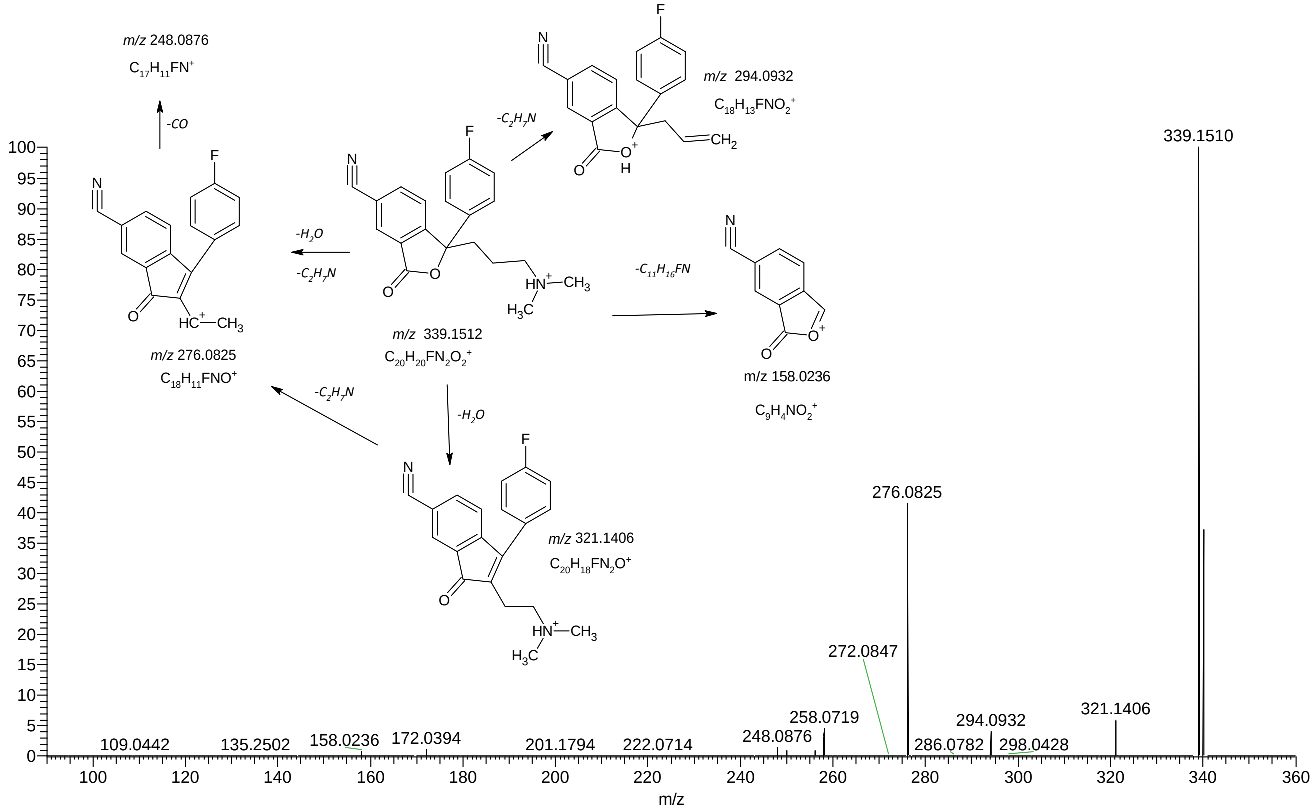

Figure S6 MS^2 spectrum of **TP339.**

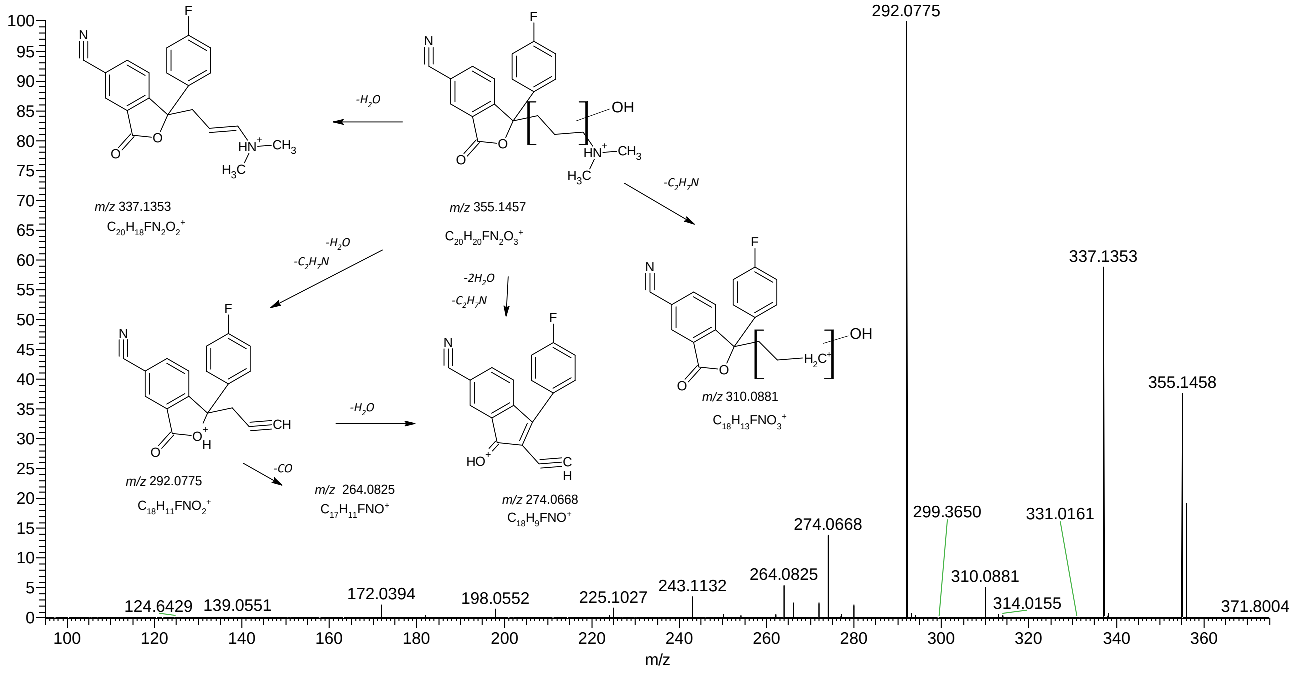

Figure S7 MS^2 spectrum of **TP355.**

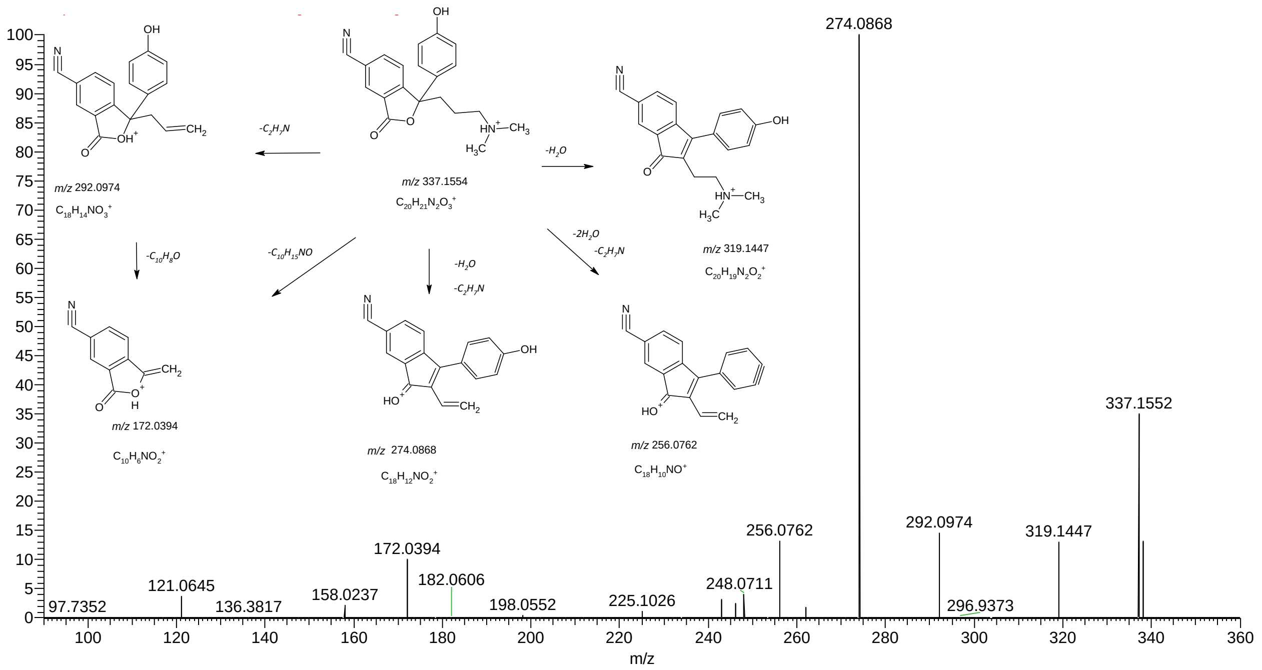

Figure S8 MS^2 spectrum of **TP337.**

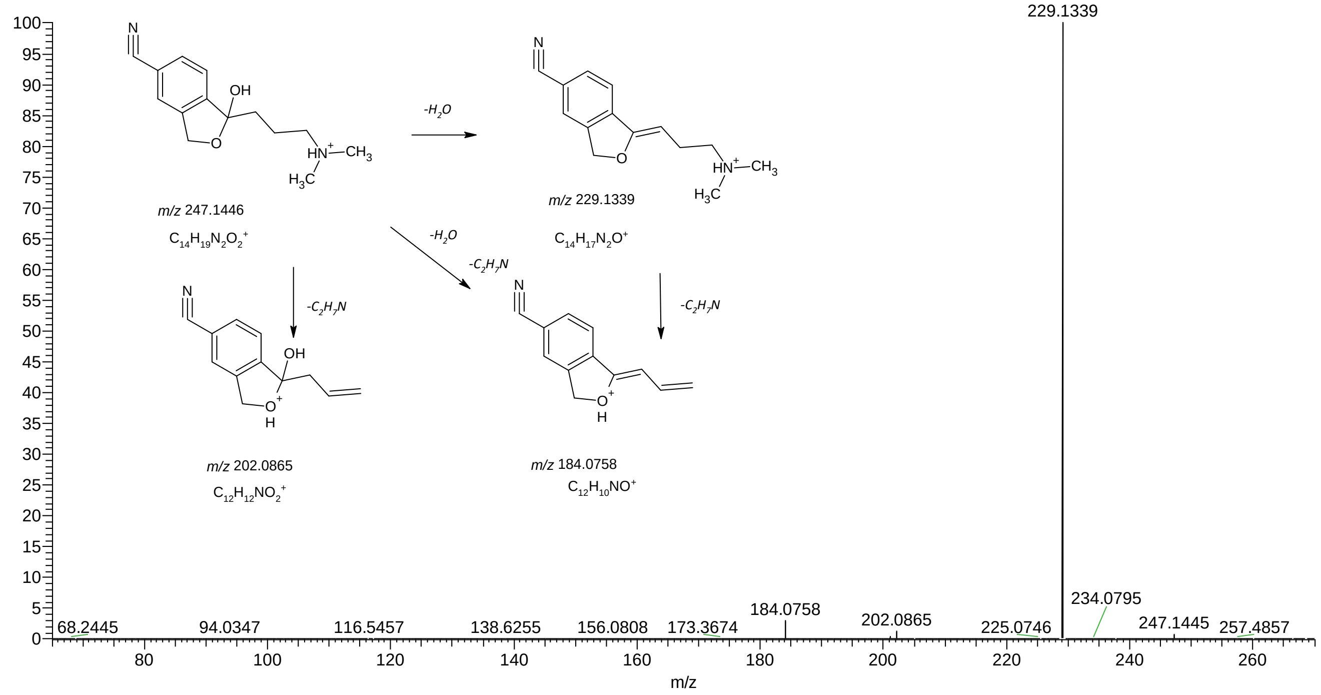

Figure S9 MS^2 spectrum of **TP247.**

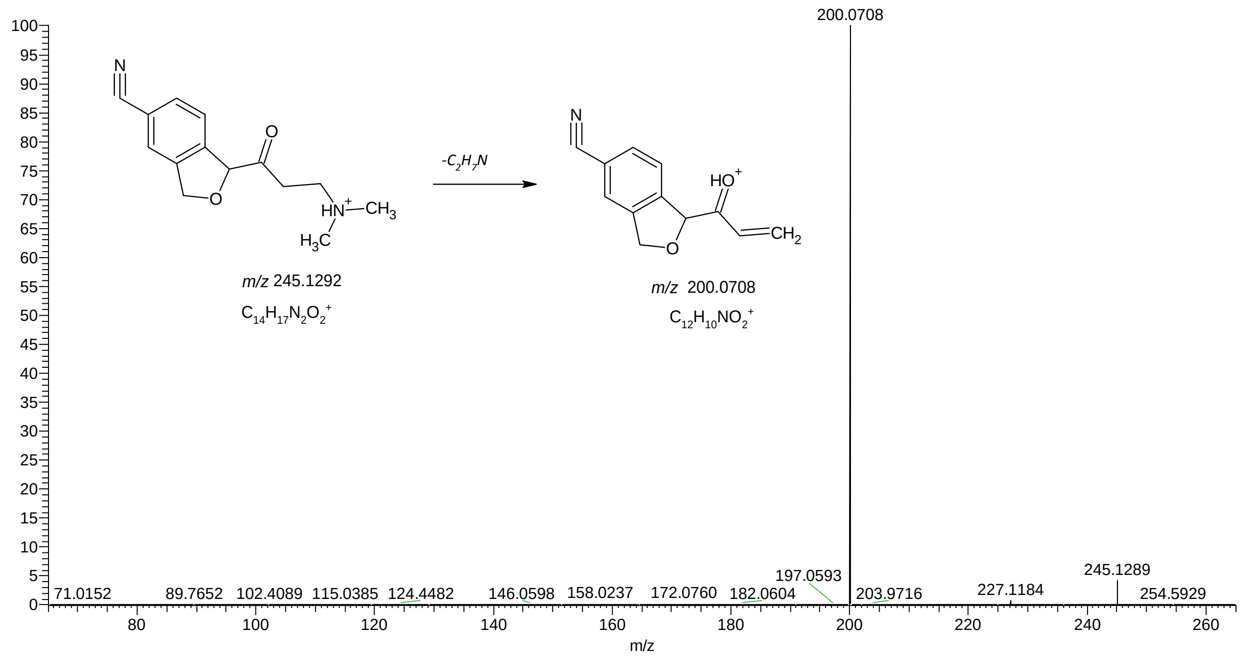

Figure S10 MS^2 spectrum of **TP245A.**

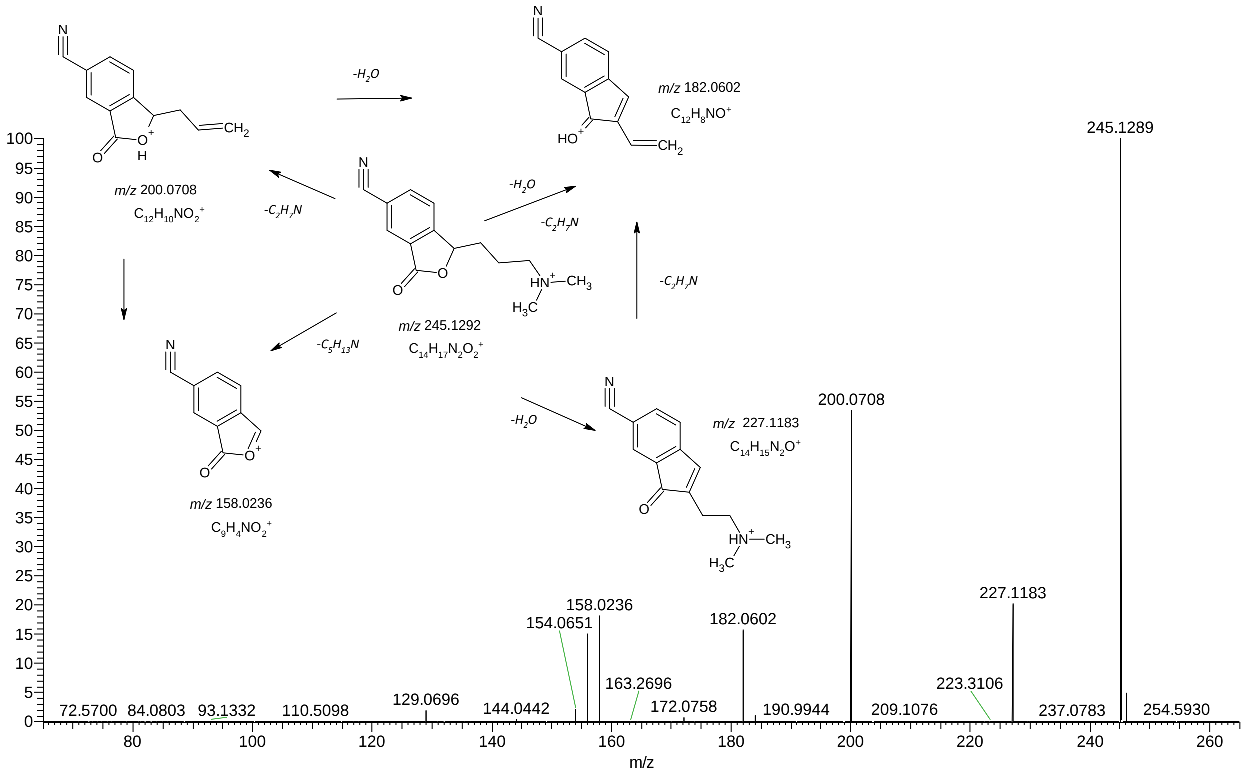

Figure S11 MS^2 spectrum of **TP245B.**

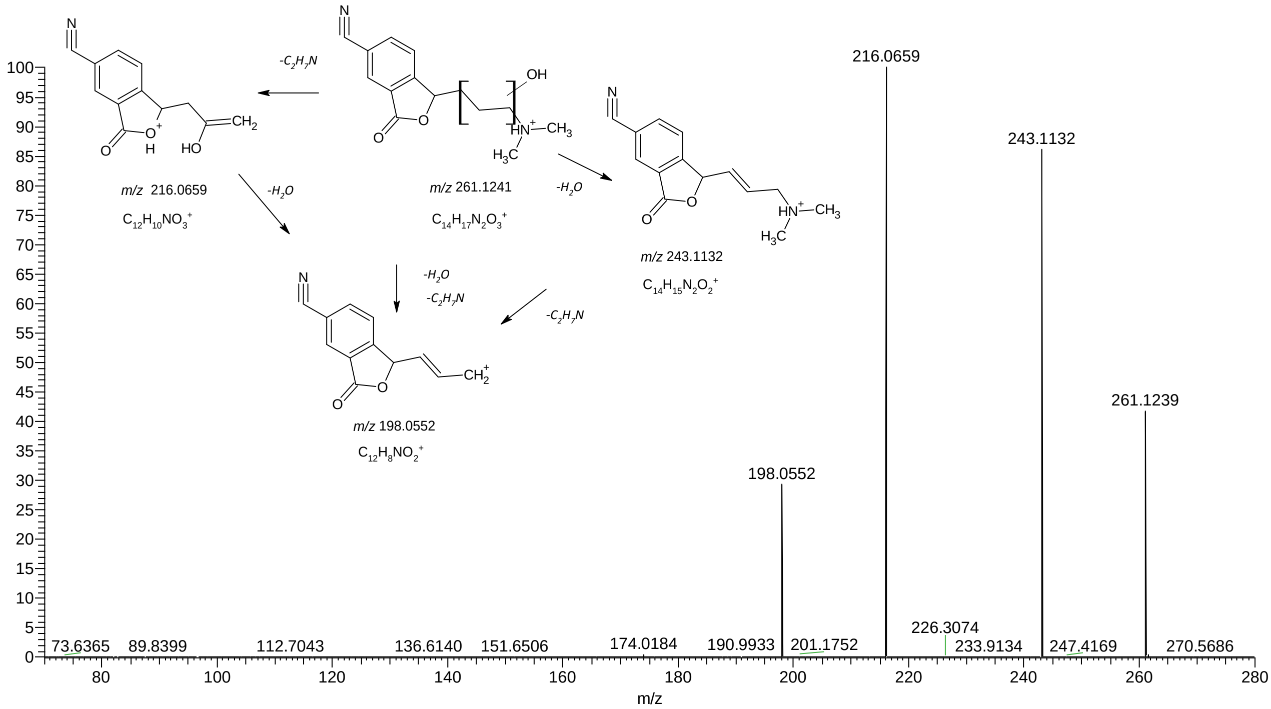

Figure S12 MS^2 spectrum of **TP261.**

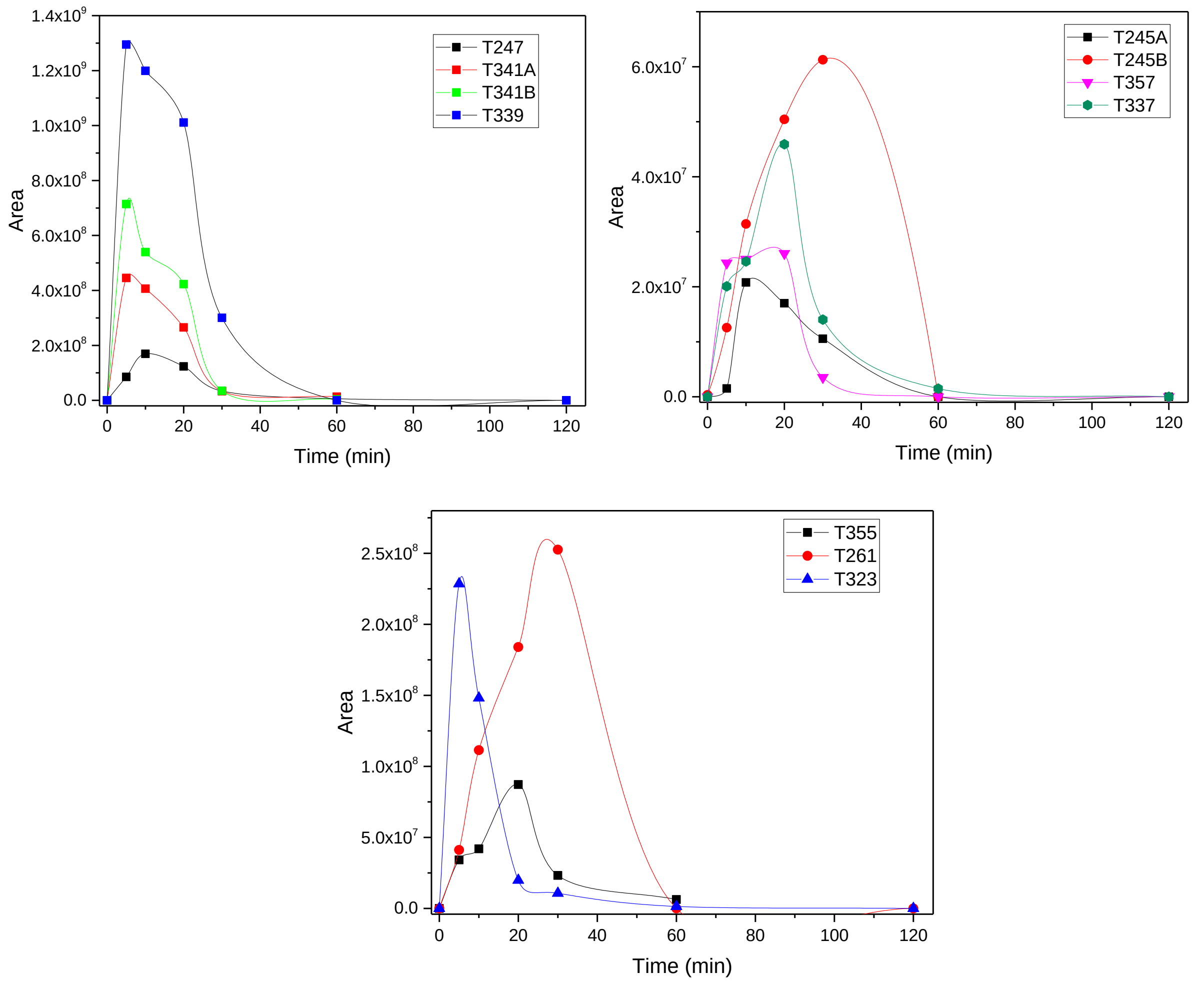

Figure S13 Time evolution of TPs detected during the photocatalytic treatment.

References

1. Osawa, R.A.; Carvalho, A.P.; Monteiro, O.C.; Oliveira, M.C.; Florêncio, M.H. Transformation products of citalopram: Identification, wastewater analysis and in silico toxicological assessment. *Chemosphere* **2019**, *217*, 858–868, doi:10.1016/j.chemosphere.2018.11.027.

2. Hörsing, M.; Kosjek, T.; Andersen, H.R.; Heath, E.; Ledin, A. Fate of citalopram during water treatment with O3, ClO2, UV and fenton oxidation. *Chemosphere* **2012**, *89*, 129–135, doi:10.1016/j.chemosphere.2012.05.024.

3. Jiménez-Holgado, C.; Sakkas, V.; Richard, C. Phototransformation of three psychoactive drugs in presence of sedimental water extractable organic matter. *Molecules* **2021**, *26*, 1–17, doi:10.3390/molecules26092466.
